# Spatio-Temporal Coupling Evolution of Urbanisation and Carbon Emission in the Yangtze River Economic Belt

**DOI:** 10.3390/ijerph20054483

**Published:** 2023-03-02

**Authors:** Huijuan Fu, Bo Li, Xiuqing Liu, Jiayi Zheng, Shanggang Yin, Haining Jiang

**Affiliations:** 1 School of Computer Science and Technology, Zhejiang Normal University, Jinhua 321004, China; 2 School of Management, Tianjin University of Technology, Tianjin 300384, China; 3 College of Geography and Environmental Sciences, Zhejiang Normal University, Jinhua 321004, China

**Keywords:** urbanisation, carbon emissions, coupling coordination degree, spatio-temporal evolution, the Yangtze River Economic Belt

## Abstract

The distribution characteristics of urbanisation level and per capita carbon emissions from 2006 to 2019 were investigated by the ranking scale rule, using 108 cities in the Yangtze River Economic Belt of China. A coupling coordination model was established to analyse the relative development relationship between the two, and exploratory spatial–temporal data analysis (ESTDA) was applied to reveal the spatial interaction characteristics and temporal evolution pattern of the coupling coordination degree. The results demonstrate that: (1) The urbanisation level and per capita carbon emissions of the Yangtze River Economic Belt show a stable spatial structure of ‘high in the east and low in the west’. (2) The coupling and coordination degree of urbanisation level and carbon emissions show a trend of ‘decreasing and then increasing’, with a spatial distribution of ‘high in the east and low in the west’. (3) The spatial structure exhibits strong stability, dependence, and integration. The stability is enhanced from west to east, the coupling coordination degree has strong transfer inertia, and the spatial pattern’s path dependence and locking characteristics show a trend of weak fluctuation. Therefore, the coupling and coordination analysis is required for the coordinated development of urbanisation and carbon emission reduction.

## 1. Introduction

With the acceleration of industrialisation and urbanisation worldwide, the emissions of greenhouse gases such as carbon dioxide are gradually increasing, rendering global warming as one of the main global concerns. According to the International Energy Agency, carbon dioxide emissions from urban energy consumption will increase at a rate of 1.8% from 2006 to 2030, while the share of cities in global carbon dioxide emissions will increase from 71% to 76% during the same period [1]. In recent years, cities have gradually become the largest sources of energy consumption and greenhouse gas emissions [2], and the impact of urbanisation on carbon dioxide emissions has received increasing attention [3,4]. China became the world’s largest carbon emitter in 2006; however, by 2019, China’s total carbon emissions accounted for 28.76% of the world’s total carbon emissions [5]. In September 2020, China proposed the target of ‘carbon peak’ by 2030 and ‘carbon neutrality’ by 2060. In 2021, ‘carbon peak’ and ‘carbon neutrality’ will be included in the government work report of the State Council, officially opening the first year of ‘dual-carbon’. Under the guidance of the ‘dual-carbon’ target, reducing the growth rate of carbon emissions while continuing to enhance the quality of urbanisation development is an important issue for China in the future. The Yangtze River Economic Belt is the economic corridor with the highest urban density in China. It is also the inland river basin economic belt with the largest development scale and widest influence range globally. Exploring the relationship between urbanisation and per capita carbon emissions in the Yangtze River Economic Belt is related to whether the Yangtze River Economic Belt can achieve low-carbon transition and green development, and affects the process of China’s carbon emissions and the achievement of the ‘carbon peak’ and ‘carbon neutrality’ goals.

As the world focuses on the carbon emissions problem, an increasing number of scholars have focused their research on carbon emissions and have achieved rich research results, specifically emphasising the influencing factors of carbon emissions [6,7], spatial and temporal evolution laws [8,9], calculation models [10,11], institutional policies for carbon emissions trading [12,13], and others. Furthermore, they have proposed reduction measures for carbon emissions for different industries [14,15]. Most scholars have used the STIRPAT model [16,17,18], Granger causality test [19], least squares method [20], regression analysis [21,22], and Kuznets curve [23,24] to study the impact of urbanisation on carbon emissions. There are differences in the relationship between urbanisation and carbon emissions owing to differences in research methods and perspectives, which can be mainly attributed to three categories: first, an increase in urbanisation promotes carbon emissions [25,26]; second, an increase in the urbanisation level has a suppressive effect on carbon emissions [27,28,29,30]; and third, there is a nonlinear relationship between urbanisation and carbon emissions, and many studies’ results show an inverted U-shaped relationship between urbanisation and carbon emissions [21,31,32,33,34].

The following problems still exist in the current research on the relationship between urbanisation and carbon emissions: (1) From the perspective of research methods, most of the existing literature focuses on time series or a dimension in the spatial pattern and very few combine temporal changes with spatial evolution, especially using spatial and temporal data analysis techniques. (2) Regarding the research content, most of the existing studies investigate the response of carbon emissions under the development process of urbanisation, but the spatial and temporal evolution of the coupling relationship between urbanisation and carbon emissions has not been emphasised. (3) Regarding the research scale, most of the existing studies focus on urban clusters with high urbanisation levels or provinces and regions with high carbon emissions. They lack research on the relationship between urbanisation and carbon emissions development in watershed-type economic zones.

Therefore, this study uses the Yangtze River Economic Belt as the study area to explore the coupling development law of urbanisation and carbon emissions, reveal the spatial and temporal evolution characteristics of the coupling coordination degree, and propose differentiated urban construction and development policies for different types of cities based on the research results. The academic contribution of this study and the expansion of the existing relevant research are mainly reflected in the following three aspects. (1) Exploring the characteristics of urbanisation level and per capita carbon emissions in the Yangtze River Economic Belt from the time and spatial level, respectively, the exploratory spatial–temporal data analysis (ESTDA) technique is used to reveal the spatial and temporal evolution of the coupling coordination degree between urbanisation level and per capita carbon emissions, thus enriching the research tools. (2) Constructing a conceptual model of the coupled development of urbanisation and per capita carbon emissions, and using various technical means to deeply reveal the spatial and temporal structural characteristics of the coupled coordination degree, not only deepens the research content of the interaction between urbanisation level and carbon emissions but also expands the research paradigm. (3) The Yangtze River Economic Belt, a basin-based economic zone, spans three major regions in China, including the Yangtze River Delta city cluster, the middle reaches of the Yangtze River city cluster, and the Chengdu–Chongqing city cluster. It is a major national strategic development region in China. Exploring the interaction between urbanisation and per capita carbon emissions in this region can not only guide the achievement of the ‘dual-carbon’ goal in the Yangtze River Economic Belt but also provide practical references for carbon reduction in urban clusters or basin-based economic zones in other countries and regions of the world.

## 2. Spatial Coupling Mechanism of Urbanisation and Carbon Emission

**Figure 1:**
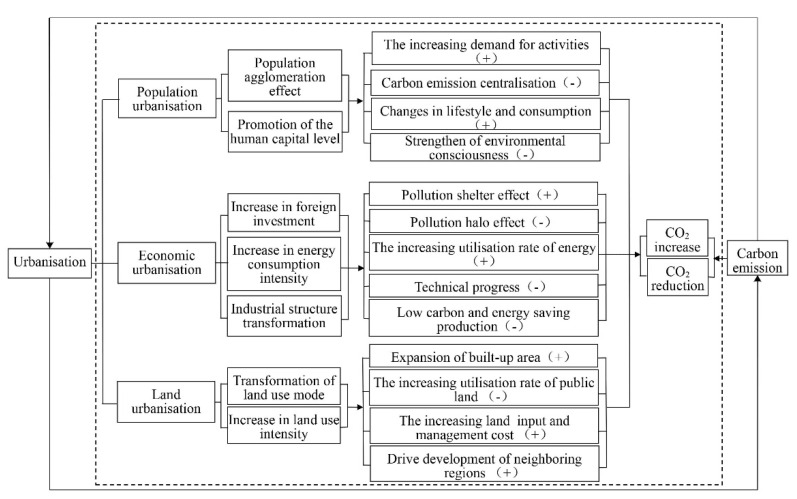
Spatial coupling mechanism of urbanisation and carbon emissions. Note: The symbol ‘+’ represents an increase in carbon emissions and ‘−’ represents a decrease in carbon emissions.

A close dynamic interaction mechanism exists between urbanisation and carbon emissions (Figure 1). Urbanisation is a complex system involving many disciplines, such as geography, economics, sociology, and ecology. Urbanisation is also a comprehensive process, which mainly manifests as an increase in the proportion of the urbanised population, the expansion of urban land scale, and advanced industrial structure; thus, it can be categorised into population, economic, and land urbanisation. Population urbanisation manifests itself in the form of the population agglomeration effect, high-density urban structure, and human capital level enhancement, which can be identified in the form of increased foreign investment, increased energy consumption intensity, and industrial structure transformation and upgrading. Land urbanisation manifests itself mainly in the form of land use transformation and land use intensity enhancement. Different activities and their forms determine whether carbon dioxide (CO_2_) emissions increase or decrease. Irrational, high-intensity, and roughly operated activities will increase CO_2_ emissions, while rational, low-intensity, and intensively produced activities will reduce CO_2_ emissions. This study attempts to analyse the coupling mechanism between urbanisation and per capita carbon emissions in the different dimensions mentioned above and clarifies the spatial coupling mechanism.

Population is the main indicator of urbanisation, and the proportion of the urban population to the total population is an important vehicle for most scholars studying the level of urbanisation. With the increasing scale of urbanisation and the continuous influx of population into cities, population has become the principal causal factor of carbon emissions growth [35]. Reasonable control of population size and improvement in population quality can contribute to improving efficiency and reducing management costs, which in turn can reduce CO_2_ emissions. The population agglomeration effect can decrease the expansion of the energy use scale caused by the dispersal of the population, which helps to centralise the emissions and treatment of CO_2_ and offsets the carbon emissions from population growth to a certain extent. The limited urban land and building structure gradually tend to be constricted with the increase in population and income in per capita car ownership. On the one hand, residents start buying smaller vehicles to ensure road capacity, reducing fuel consumption. On the other hand, the increase in per capita car ownership tends to cause traffic congestion, thus creating a ‘stop-and-go’ driving pattern [36], exacerbating the emissions of hydrocarbons and other gases. The rich urban resources provide the conditions for the cultivation of talent, and the excellent educational resources raise the environmental awareness of citizens. With the infiltration of urban concepts, residents’ lifestyles and consumption patterns gradually move from being slow-paced to fast-paced, which increases carbon emissions. Simultaneously, the demand for energy and electricity increases.

Urbanisation is a process of social and economic transformation, not only in the transformation from an agricultural to an urban population but also in the transformation of an agricultural economy to an industrial and service economy. The increase in the urbanisation level depends on economic development, so the development pattern of the urban economy directly affects the level of carbon emissions. The increase in foreign investment can usher new activities into the development of cities but have positive and negative effects on carbon emissions [37]. When the introduced foreign investment is high pollution, high emissions, and high energy-consuming traditional industries, it will produce the ‘pollution shelter effect’, that is, will increase carbon emissions. However, when green, energy-saving, and high-efficiency new industries are introduced, the ‘pollution halo effect’ is generated, reducing carbon emissions in the introduced areas and improving the air quality level. The increase in urbanisation level and economic competition between regions stimulates the transformation and upgrading of industrial structures. That means eliminating traditional industries and shifting to more low-carbon, clean, and energy-saving industries [38], reducing resource dependence, and reducing carbon emissions to achieve ecologically sustainable urban development. The transformation and upgradation of the industrial structure further stimulate the demand for energy consumption, and the increase in residential electricity and energy consumption has a counterproductive effect on reducing carbon emissions. With the increase in energy consumption intensity, the advancement of energy-saving technologies and the replacement of renewable energy sources guarantee urban development [39] while positively affecting the reduction of carbon emissions.

The differences in land use patterns reflect changes in the scale of urban development, and the expansion of the area of nonagricultural land, in general, implies the expansion of the scale of urban development. The change in urban land use type is reflected in the encroachment of many other types of land, especially agricultural and natural forest land, resulting in the expansion of built-up areas and the reduction in agricultural and natural forest land. As a ‘carbon sink’, reducing the natural woodland area also increases CO_2_ emissions. Meanwhile, owing to the proximity effect, urbanisation will drive the economic and social development of the surrounding areas, further promoting the carbon emissions of neighbouring areas [40]. Although some cities have gradually focused on greening the landscape at a certain stage of development, the effect on carbon emissions reduction is not significant because of the high cost of artificial greening input management [41,42]. The optimal management of existing built-up areas can help improve the efficiency of public land use and slow encroachment on other types of land, which indirectly reduces carbon emissions.

## 3. Data Sources and Methods

### 3.1. Study Area and Data Sources

#### 3.1.1. Study Area

The Yangtze River Economic Belt spans across three regions—eastern, western, and central China—covering a wide range of areas, with large differences in socioeconomic conditions, urbanisation development levels, and ecological development in the region. It consists of 11 provincial administrative units in Shanghai, Jiangsu, Zhejiang, Anhui, Jiangxi, Hubei, Hunan, Chongqing, Sichuan, Guizhou, and Yunnan. Thus, the Yangtze River Economic Belt is one of the three major strategies implemented by China: It is an inland economic zone with global influence; a coordinated development zone with interaction and cooperation through east, west, and central China; an open zone for domestic and foreign trade along the coast, rivers, and borders; and an early demonstration zone for ecological civilisation.

#### 3.1.2. Data Sources

**Figure 2:**
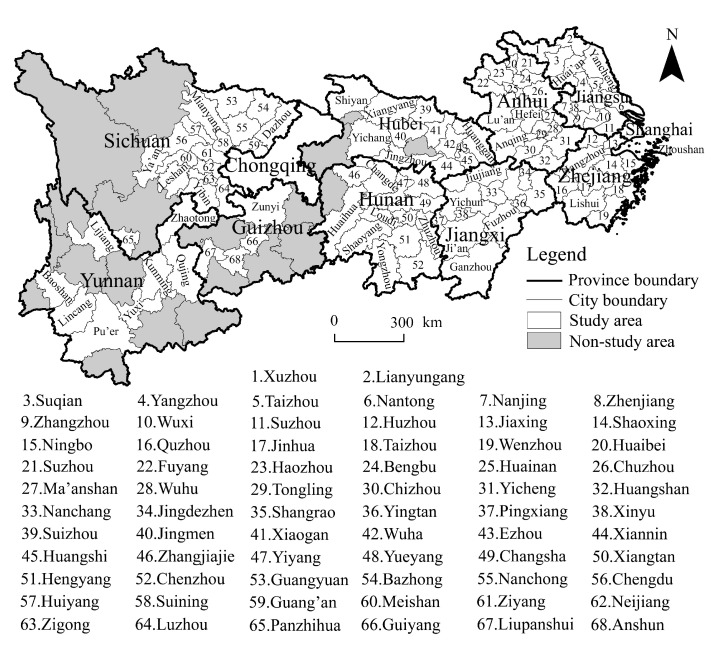
Study area.

According to the 2019 administrative division, the Yangtze River Economic Belt contains two municipalities directly under the central government, 108 prefecture-level cities, and 16 autonomous prefectures, totalling 126 cities at the prefecture level and above (Figure 2). This study categorises urbanisation into three core subsystems: population, land, and economy. It uses the proportion of urban population to resident population to characterise population urbanisation, the proportion of built-up areas to municipal areas to characterise land urbanisation, and the proportion of added value of tertiary industry to GDP to characterise economic urbanisation and adopts these three indicators to construct the comprehensive development level of urbanisation [43,44,45]. Given that China became the world’s largest carbon emitter in 2006, the urbanisation index data for this study were mainly obtained from the 2007–2020 China Urban Statistical Yearbook, the China Urban Construction Statistical Yearbook, and the statistical yearbooks of provinces and cities. The carbon emissions data were obtained by fitting the training DMSP/OLS and NPP/VIRRS data with the particle swarm optimisation–back propagation (PSO-BP) algorithm of Chen et al. [46], which was verified by energy accounting, and the goodness of fit was as high as 0.988, which is highly reliable and significantly better than other carbon emissions data. The carbon emissions data of 108 cities from 2006 to 2019 were obtained by combining the carbon emissions of counties in the CEADs database (www.ceads.net). Per capita carbon emissions were calculated by dividing total urban carbon emissions by the total population at the end of the year, which was derived from the China City Statistical Yearbook 2007–2020.

### 3.2. Research Methods

#### 3.2.1. Measurement of Urbanisation Level

Urbanisation is a comprehensive process that includes population, land, and economy. Using comprehensive indicators to measure urbanisation subsystems can reflect comprehensive development; however, there may be multiple covariates, and this cannot highlight specific important factors or key indicators. Therefore, this study categorises urbanisation into three core subsystems of population, land, and economy and adopts these three indicators to construct the comprehensive development level of urbanisation. The entropy method can objectively and truly reflect the effective information implicit in the index data and reduce the subjectivity of evaluation [47,48]; therefore, it is used to determine the weights of the three indicators, which were standardised using the extreme difference method, and the information entropy and weights of each indicator were subsequently calculated to obtain the weights of the proportion of resident population, the proportion of built-up area, and the proportion of the three industries as 0.3965, 0.2532, and 0.3503, respectively. The weighted summation method was used to calculate the comprehensive urbanisation development level of each city in the Yangtze River Economic Belt using the following formula:

(1) $$UL_{i}=\sum_{j=1}^{3}Z_{ij}\times w_{j}$$

where *UL_i_* is the comprehensive urbanisation development level of city *i*; *Z_ij_* is the standardised value of variable *j* in city *i*; and *w_j_* is the weight of variable *j*.

#### 3.2.2. Coupling Coordination Degree Model

Coupling is a physical concept that refers to the phenomenon of mutual influence between two or more systems or forms of motion through various interactions [49]. Coordination is a benign interrelationship between two or more systems or elements and is a harmonious, consistent, and virtuous cycle between systems or elements within systems [50,51]. In this study, the degree of mutual influence of two elements, urbanisation level and per capita carbon emissions, is defined as the degree of coupling coordination. It consists of three components: the development degree (*T*), coupling degree (*C*), and coordination degree (*D*). 

(1) Development degree: This refers to the comprehensive evaluation index that reflects the overall benefits of urbanisation level and per capita carbon emissions and its expression is as follows:

(2) $$T=a\mu_{1}+b\mu_{2}$$

In this formula, *a* and *b* are the weights to be determined, considering that in the process of social development, the improvement of urbanisation development level and reduction of carbon emissions are equally significant, so *a* and *b* are both taken as 0.5; *μ*_1_ and *μ*_2_ are the standardised values of urbanisation development level and per capita carbon emissions.

(2) Coupling degree: This refers to the coupling degree of urbanisation development level and per capita carbon emissions and measures the degree of interdependence between the two, which is expressed as follows:

(3) $$C=m{\left\{\left(\mu_{1}\times \mu_{2}\times \cdot \cdot \cdot \times \mu_{m}\right)/{\left(\mu_{1}+\mu_{2}+\cdot \cdot \cdot +\mu_{m}\right)}^{m}\right\}}^{1/m}$$

In this formula, *μ* (*n* = 1, 2, …, *m*) is the evaluation value of each subsystem, and *m* is the number of subsystems. As this study focuses on the level of urbanisation development and per capita carbon emissions of two subsystems, *m* = 2. C is the coupling degree, and 0 ≤ *C* ≤ 1; the larger the value of *C*, the more coupled the level of urbanisation development and per capita carbon emissions reduction, and vice versa.

(4) $$C=2{\left\{\left(\mu_{1}\times \mu_{2}\right)/{\left(\mu_{1}+\mu_{2}\right)}^{2}\right\}}^{1/2}$$

(3) Coordination degree: It is a quantitative indicator to measure the status of coordinated development, integrating coupling degree *C* and development degree *T* with high stability, reflecting the relationship between urbanisation development level and per capita carbon emissions from disorderly to orderly development trends, whose expression is as follows:

(5) $$D=\sqrt{C\times T}$$

#### 3.2.3. Exploratory Spatial–Temporal Data Analysis

The concept of ESTDA was introduced to reveal the spatial and temporal structural characteristics of the urbanisation level of the Yangtze River Economic Belt and the coupling coordination degree of per capita carbon emissions, systematically analysing the spatial interaction characteristics and the law of spatial and temporal evolution in the spatial and temporal evolution processes. Furthermore, ESTDA effectively compensates for the shortage of ESDA detection in the time dimension and realises the benign coupling of spatio-temporal and spatial measures [5], which mainly includes analysis techniques, such as local indicators of spatial association (LISA) time path and spatio-temporal leap. 

(1) LISA time path. Observing the evolution characteristics of the LISA in the time dimension for each cell in the Moran scatter plot makes the static LISA more dynamic [52]. By visualising the pairwise movement of the coupling coordination degree and its spatial lag term, the spatio-temporal synergistic evolution of the coupling coordination degree of the urbanisation level and per capita carbon emissions intensity in the Yangtze River Economic Belt can be explained, and the spatio-temporal dynamics of local spatial differences and changes in the coupling coordination degree can be reflected. The indicators of the LISA time path include path length, curvature, and leap direction. The LISA time path length can reflect the dynamic characteristics of the local spatial structure of the coupling coordination degree. The curvature reflects the fluctuation characteristics of the coupling coordination degree’s local spatial structure, and the leap direction reflects the integration characteristics of the evolution of the local spatial structure of the coupling coordination degree. The specific expressions are as follows [53]:

(6) $$d_{i}=\frac{N\sum_{t=1}^{T-1}d(L_{i,t},L_{i,t+1})}{\sum_{i=1}^{N}\sum_{t=1}^{T-1}d(L_{i,t},L_{i,t+1})}$$

(7) $$\varepsilon_{i}=\frac{\sum_{t=1}^{T-1}d(L_{i,t},L_{i,t+1})}{d(L_{i,t},L_{i,T})}$$

(8) $$\theta_{i}=\arctan\frac{\sum_{j}\sin\theta_{j}}{\sum_{j}\cos\theta_{j}}$$

where *d_i_* and *ε_i_* are the path length and the curvature of city *i*, respectively; *N* is the number of study units; *T* is the length of study time; *L_i_*_,*t*_ are the LISA coordinates of city *i* at time *t*; *d* (*L_i_*_,*t*_, *L_i_*_,*t*+1_) is the distance city *i* moves from time *t* to *t* + 1; and *θ_i_* denotes the average moving direction of city *i*.

(2) LISA spatio-temporal leap. The LISA spatio-temporal leap can reveal the spatial relationship between local neighbours of spatial units in a time-varying condition [54] and is divided into four types (Table 1): Type I indicates that the city leaps itself and the neighbouring city is stable; Type II indicates that the city is stable and the neighbouring city leaps; Type III indicates that both the city and the neighbouring city leap, Type IIIA indicates that the city and the neighbouring city leap in the same direction and Type IIIB indicates that the city and the neighbouring city leap in opposite directions; Type IV indicates that the city and the neighbouring city are stable. Rey defined the regional system of spatio-temporal flow, and coalescence was defined as the ratio of the number of leaps of a certain type to the total number of leaps during the study period [55], which can be expressed as follows:

(9) $$\mathrm{Space}–\mathrm{time}\;\mathrm{flow}\;(SF):\;SF=\frac{F_{1}+F_{2}}{m}$$

(10) $$\mathrm{Space}–\mathrm{time}\;\mathrm{condensation}\;(SC):\;SC=\frac{F_{3A}+F_{4}}{m}$$

where *F_1_*, *F_2_*, *F_3A_*, and *F_4_* are the numbers of leaps of I, II, IIIA, and IV, respectively; *m* is the total number of leaps.

## 4. Results

### 4.1. Distribution Characteristics of Urbanisation Level and Carbon Emission

**Figure 3:**
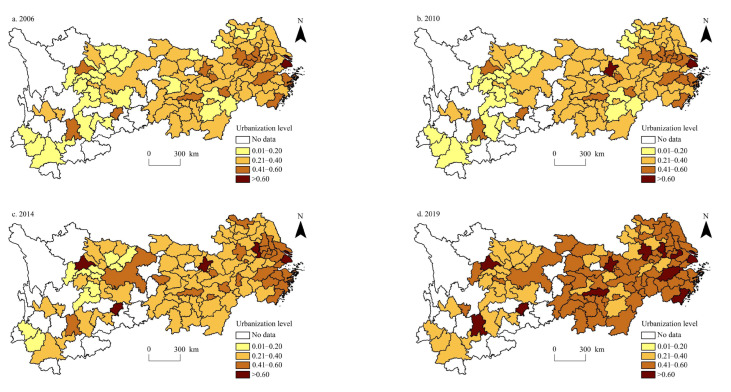
Spatial distribution pattern of urbanisation level in the Yangtze River Economic Belt during 2006–2019.

**Figure 4:**
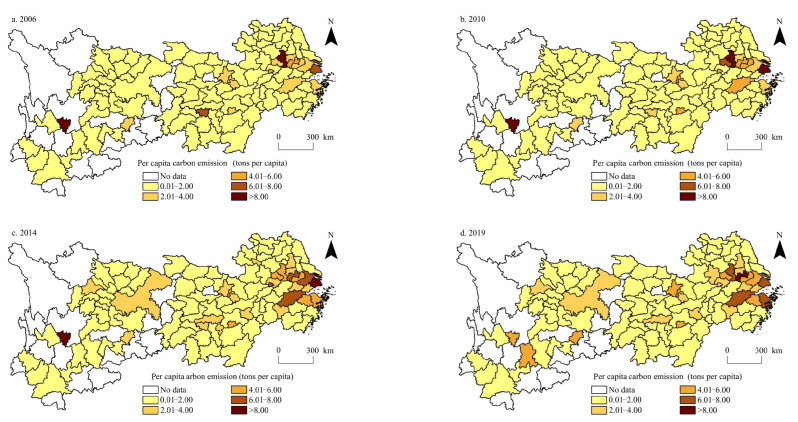
Spatial distribution pattern of per capita carbon emissions in the Yangtze River Economic Belt during 2006–2019.

The urbanisation level of cities in the Yangtze River Economic Belt was calculated from 2006 to 2019. The spatial distribution of the urbanisation level and per capita carbon emissions in the Yangtze River Economic Belt during 2006, 2010, 2014, and 2019 was visualised using the ArcGIS 10.2 software (Figure 3 and Figure 4).

Regarding the urbanisation level, the spatial patterns in 2006, 2010, 2014, and 2019 were relatively stable, showing a decreasing spatial trend from east to west (Figure 3). The high-value urbanisation areas are mainly distributed in Shanghai, southern Jiangsu, central Zhejiang, central Anhui, and other regions in the Yangtze River Delta urban agglomeration, as well as in the capital cities of other provinces, such as Wuhan, Changsha, Chengdu, Guiyang, Kunming, and Nanchang. Low-value areas of urbanisation are mainly distributed in northern Anhui, western Jiangxi, northern Hubei, northern Hunan, eastern Sichuan, western Guizhou, and southern Yunnan, while northern Jiangsu gradually transitions from low-value to medium- to high-value areas. The average urbanisation levels in 2006, 2010, 2014, and 2019 were 0.2773, 0.2985, 0.3437, and 0.4531, respectively. In other words, the urbanisation level of the Yangtze River Economic Belt overall showed an accelerating trend, and the urbanisation level of all cities increased at different amplitudes. Urbanisation levels in Yunnan, Sichuan, Guizhou, and Jiangxi increased rapidly. This is mainly owing to the poor population agglomeration effect, low level of industrial structure, and slow urban–land expansion in these regions at the beginning of the study. After 14 years of development, the urbanisation level in all dimensions greatly improved. The increase in urbanisation levels in Shanghai, Zhejiang, Hubei, and Chongqing was relatively low, mainly owing to high urban density development, industrial structure level, and the opening-up of these regions in the early stage of the study. However, these factors improved slowly in the later stage of the study, which restricted the rapid progress of urbanisation.

Regarding the per capita carbon emissions, the spatial pattern in 2006, 2010, 2014, and 2019 was also relatively stable (Figure 4). The high-value per capita carbon emissions areas are mainly cities with higher industrial development levels, which are clustered in Shanghai, southern Jiangsu, and northern Zhejiang in the Yangtze River Delta urban agglomeration and dispersed in Panzhihua in Sichuan Province. The second-highest value areas are mainly provincial capitals, such as Hefei, Nanchang, Changsha, Guiyang, Kunming, and other cities. There are many cities in low-carbon per capita emissions areas, and their spatial distribution is wide, mainly clustered in northern and western Anhui, northern and southern Jiangxi, northern Hubei, western and southern Hunan, eastern Sichuan, and southern Yunnan. The average per capita carbon emissions in 2006, 2010, 2014, and 2019 were 1.131, 1.386, 1.572, and 1.664, respectively, indicating that the overall per capita carbon emissions in the Yangtze River Economic Belt are rising. By comparing the spatial patterns of per capita carbon emissions in 2006 and 2019, it was found that there were 99 cities with rising per capita carbon emissions, accounting for 91.66% of the study area. Among them, Guizhou, Yunnan, Jiangsu, Anhui, and other regions have a rapid growth rate of per capita carbon emissions, mainly because of the gradual increase in economic activities in these regions, the improvement of the energy utilisation rate, and the continuous expansion of urban built-up areas. Furthermore, it is greatly affected by the ‘Pollution shelter effect’. Only nine cities, Nanjing, Tongling, Xiangtan, Zigong, Panzhihua, Meishan, Yongzhou, Huaihua, and Liupanshui, experienced a decrease in per capita carbon emissions. Most of these are industrial cities. Owing to technological progress and the development of low-carbon and energy-saving production, urban industrial structures have been transformed and upgraded, and per capita carbon emissions have gradually decreased.

By comparing the spatial pattern of urbanisation level and per capita carbon emissions, we found that the spatial pattern of the two has high stability, showing a distribution trend of ‘high in the east and low in the west’, and the spatial pattern is becoming increasingly similar. This is mainly owing to the close relationship between the urbanisation level and economic development activities, which are the main factors affecting per capita carbon emissions. Therefore, the correlation between the urbanisation level and per capita carbon emissions is increasing [56]. The difference is that with the improvement of urbanisation level, the difference between urbanisation levels of cities in the Yangtze River Economic Belt is gradually reduced, whereas with the increase in per capita carbon emissions, the difference between per capita carbon emissions of cities has gradually expanded, which is mainly owing to the influence of industrial transformation and upgrading. Some cities with high urbanisation levels transfer part of the traditional industries with high energy consumption and pollution to cities with relatively low urbanisation levels, resulting in the spillover effect of per capita carbon emissions [57].

### 4.2. Spatio-Temporal Coupling Evolution of Urbanisation and Carbon Emissions

#### 4.2.1. Coupling Coordination Degree of Urbanisation and Per Capita Carbon Emissions

**Figure 5:**
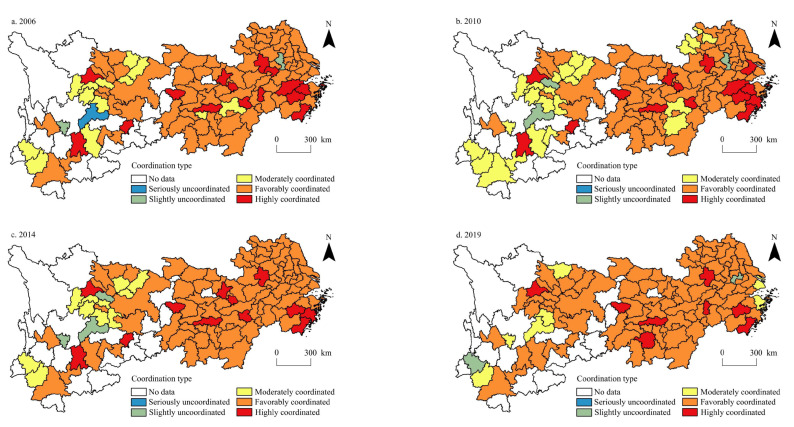
Spatial distribution of the type of coupled coordination between urbanisation level and per capita carbon emissions in the Yangtze River Economic Belt during 2006–2019.

The extreme value method was selected by standardising the data on urbanisation level and per capita carbon emissions. The coupling coordination degree correlation formula (Equations (4)–(7)) was used in this study to calculate the degree of coupling coordination between the urbanisation level and per capita carbon emissions of cities in the Yangtze River Economic Belt from 2006 to 2019. Referring to existing studies [58,59], this study divides the coupling coordination degree into five categories: severe dissonance (0.00~0.20), borderline dissonance (0.21–0.40), moderate coordination (0.41–0.60), good coordination (0.61–0.80), and high coordination (0.81–1.00). Owing to space constraints, this study only visualises 2006, 2010, 2014, and 2019 (Figure 5).

In 2006, in the Yangtze River Economic Belt, only Zhaotong in Yunnan Province, Nanjing in Jiangsu Province, and Panzhihua in Sichuan Province were among the cities with serious imbalances in urbanisation levels and per capita carbon emissions. There were 14 moderately coordinated cities, all distributed in a small number of regions in Sichuan, Yunnan, and Hunan, and 76 well-coordinated cities, which accounted for 70.37% of the study area. They are widely distributed in central and northern Jiangsu, northern and southern Anhui, northern and southern Jiangxi, Hunan, Hubei, Chongqing, northeastern Sichuan, and other regions. There were 15 highly coordinated cities, accounting for 13.88% of the study area. They were clustered in western Hunan, central and eastern Zhejiang, and central Anhui. In addition, cities with a higher level of economic development (mainly provincial capital), such as Wuhan, Changsha, Nanchang, Chengdu, Guiyang, and Kunming, also belonged to the highly coordinated type.

In 2010, Ziyang in Sichuan Province was the only city with a serious imbalance of urbanisation level and per capita carbon emissions in the Yangtze River Economic Belt; Shanghai and Nanjing in Jiangsu Province were the only two cities on the verge of imbalance. In addition, the number of moderately coordinated cities increased to 24, accounting for 22.22% of the study area, which were mainly distributed in western Yunnan, eastern Sichuan, Jiangxi, and Anhui provinces. The number of well-coordinated cities decreased to 68, accounting for 62.96% of the study area, mainly distributed in central and northern Jiangsu, Anhui, northern and eastern Jiangxi, Hubei, Hunan, Chongqing, and other regions, and 13 highly coordinated cities, accounting for 12.03% of the study area. The number and distribution range of cities were roughly the same as in 2006.

In 2014, only Ziyang in Sichuan Province had serious imbalances in urbanisation level and per capita carbon emissions in the Yangtze River Economic Belt, and the cities on the verge of imbalance were Zhaotong in Yunnan and Panzhihua in Sichuan. The number of moderately coordinated cities was reduced to 14, all of which were distributed in the western part of the Yangtze River Economic Belt, such as southwest Yunnan, northeast, and southeast Sichuan. The number of well-coordinated cities increased to 81, accounting for 75% of the study area, mainly distributed in Jiangsu, Anhui, Jiangxi, Hubei, Hunan, Chongqing, Guizhou, eastern Sichuan, and other regions. Furthermore, 10 highly coordinated cities, accounting for 9.25% of the study area, were mainly distributed in central and eastern Zhejiang and provincial cities with higher economic development levels.

In 2019, there were no cities with serious imbalances in the urbanisation level and per capita carbon emissions in the Yangtze River Economic Belt. Two cities were still on the edge of imbalance—Changzhou in Jiangsu Province and Baoshan in Yunnan Province—and the number of moderately coordinated cities was reduced to nine: Luzhou, Panzhihua, Yibin, and Guangyuan in Sichuan Province; Shanghai and Ningbo in Zhejiang Province; Ezhou in Hubei Province; and Zhaotong and Lincang in Yunnan Province. The number of well-coordinated cities increased to 89, accounting for 82.40% of the study area. They mainly clustered in central and northern Jiangsu, northern and eastern Zhejiang, Anhui, Jiangxi, Hubei, Hunan, Chongqing, Sichuan, Guizhou, and other regions, and the number of highly coordinated cities reduced to eight, accounting for 7.40% of the study area. The spatial distribution is relatively dispersed, the cities are mainly cities with high economic development levels, and many provincial capitals are the main ones.

From the perspective of time variation of the coupling coordination degree, the average coupling coordination degree between urbanisation level and per capita carbon emissions in the Yangtze River Economic Belt in 2006, 2010, 2014, and 2019 was 0.6992, 0.6848, 0.6910, and 0.6943, respectively. The average degree of coupling coordination generally showed a trend of first decreasing and then increasing. From the perspective of the spatial pattern of the coupling coordination degree, the distribution trend is ‘high in the east and low in the west’, and the spatial pattern has strong stability in time. Among them, Changzhou in Jiangsu Province and Baoshan in Yunnan Province are listed as cities on the verge of imbalance, though the reasons for their imbalance are different. The increase in per capita carbon emissions caused by the industrial agglomeration effect and energy consumption intensity in Changzhou is significantly greater than the benefits brought by urbanisation development, and the coupling coordination degree between per capita carbon emissions and urbanisation level is low. As a member of the construction of industrial key towns in western Yunnan, Baoshan has a low urbanisation level due to geographical location and environmental restrictions, so the coupling coordination degree between per capita carbon emissions and urbanisation level is low. From the perspective of spatial differences in coupling coordination degree, the standard deviations in 2006, 2010, 2014, and 2019 were 0.1137, 0.1217, 0.1134, and 0.0958, respectively, and the standard deviations showed a trend of rising and then decreasing; that is, with an increase in coupling coordination degree, the spatial difference of coupling coordination degree in the Yangtze River Economic Belt decreased.

#### 4.2.2. LISA Time Path Analysis of Coupling Coordination Degree of Urbanisation Level and Per Capita Carbon Emissions

**Figure 6:**
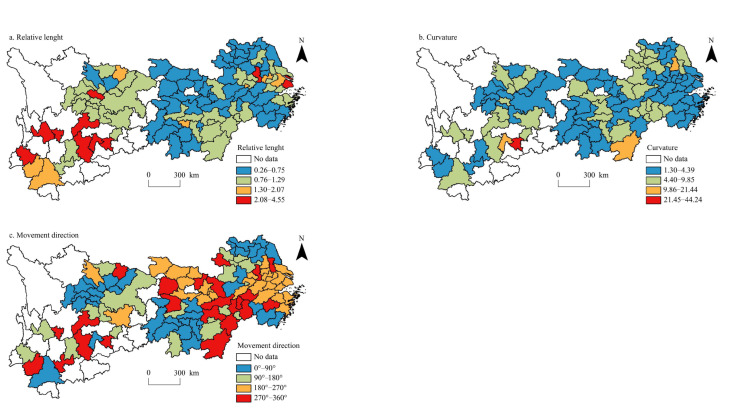
Spatial distribution of LISA time path of the coupling coordination between urbanisation level and per capita carbon emissions in the Yangtze River Economic Belt.

The relative length, curvature, and moving direction of the coupling coordination degree between urbanisation level and per capita carbon emissions in the Yangtze River Economic Belt were obtained using the LISA time path calculation formula and visualisation (Figure 6).

The relative length of the coupling coordination degree between the urbanisation level and Yangtze River Economic Belt per capita carbon emissions presents a spatial distribution pattern of ‘high in the west and low in the east’. The cities with high relative lengths are mainly distributed in Yunnan, Guizhou, Sichuan, Chongqing, and Jiangxi. Shanghai, Hefei, Nanjing, and other municipalities directly under the Central Government or provincial capitals also have high relative lengths, indicating that the degree of coupling coordination in these regions varies greatly. The cities with low relative lengths are mainly distributed in Zhejiang, Hubei, southern Jiangsu, and northern Anhui, which reflects the small change in the coupling coordination degree in these regions. There are 76 cities in the Yangtze River Economic Belt, where the relative length of the coupling coordination degree is lower than 1 (i.e., the average), accounting for 70.37% of the study area. This indicates that the spatial structure of the coupling coordination degree of the Yangtze River Economic Belt has strong stability, and this stability gradually increases from west to east. This is mainly due to the high level of economic development and scientific and technological development in the eastern region of the Yangtze River Economic Belt, which leads to a high level of carbon emission reduction technology development. Therefore, the urban level and per capita carbon emission are promoted cooperatively, so the coupling coordination degree is relatively stable. However, the urbanisation level in western China is rapidly improved, the intensity of economic activities is gradually enhanced, and the level of industrial structure is relatively low, which accelerate the growth of per capita carbon emissions. Therefore, the coupling coordination degree rises rapidly, that is, it has strong variability. In the Yangtze River Economic Belt, the coupling coordination degree curvature of the urbanisation level and per capita carbon emissions is generally low, and the distribution of high-value areas is relatively dispersed. For example, in Anshun, Liupanshui, Ganzhou, Yangzhou, and other regions, the bending degree of these cities is more than 14, and the bending degree of Anshun is the highest, reaching 44.23, indicating that the local spatial dependence direction of the coupling coordination degree of these cities is highly variable. Cities with low curvature are numerous and widely distributed, mainly clustered in Zhejiang, Hubei, Hunan, Chongqing, northern Jiangsu, southern Anhui, and central Sichuan, indicating that the fluctuation in the coupling coordination degree of these cities is small. In general, the curvature of the coupling coordination degree in the Yangtze River Economic Belt is mostly maintained at a low level, which reflects that the evolution of the coupling coordination degree between urbanisation level and per capita carbon emissions presents a relatively stable spatial dependence; that is, the coupling coordination degree has a strong spatial locking effect. Under the influence of many factors, such as the population agglomeration effect, high-density urban structure, industrial structure transformation, and land-use change, the coupling coordination degree of cities in the Yangtze River Economic Belt presents a solid, stable dependence.

Regarding the moving direction, there are 66 cities in the Yangtze River Economic Belt, where the urbanisation level and coupling coordination degree of per capita carbon emissions increase in synergy (0°–90° and 180°–270°), accounting for 61.11% of the study area, indicating that the spatial evolution of the coupling coordination degree presents strong spatial integration. There were 37 cities with positive collaborative growth (0°–90°), accounting for 56.06% of the cities with collaborative growth, mainly distributed in central and northern Jiangsu, southern Zhejiang, southern Hunan, central Sichuan, and other regions. The degree of coupling coordination in these regions demonstrates the characteristics of collaborative high-speed growth. This is mainly owing to the accelerated improvement of the urbanisation level in these regions and the increase in per capita carbon emissions owing to the enhancement of the population agglomeration effect, economic activity intensity, and energy consumption intensity. There were 29 cities with negative collaborative growth (180°–270°), accounting for 43.93% of the cities with collaborative growth, mainly concentrated in Shanghai, southern Jiangsu, northern Zhejiang, Hubei, and other regions. The degree of coupling coordination in these regions showed characteristics of low collaborative growth. Most of these regions are cities with a high level of economic development, and their urbanisation levels are advancing steadily. Owing to the concentration of carbon emissions, the improvement of energy use efficiency, and the improvement of low-carbon and energy-saving technologies, the growth rate of per capita carbon emissions decreases, so the coupling coordination degree shows collaborative low-speed growth.

#### 4.2.3. LISA Spatio-Temporal Transition Analysis of Coupling Coordination Degree of Urbanisation Level and Per Capita Carbon Emissions

Based on the probabilistic transfer matrix and spatio-temporal transition, the transfer characteristics and evolution process of local spatial correlation types of urbanisation level and coupling coordination degree of per capita carbon emissions in the Yangtze River Economic Belt were analysed (Table 2).

The probability of Moran’s *I* scatter plot remaining in the same quadrant (Type IV) was above 86% in three subperiods from 2006 to 2010, 2010 to 2014, and 2014 to 2019 and in the study period from 2006 to 2019. The results show strong transfer inertia between the urbanisation level and coupling coordination degree of per capita carbon emissions in the Yangtze River Economic Belt. The coupling coordination degree types of cities have strong stability, and the spatial pattern of coupling coordination degree has strong path dependence and spatial locking characteristics. The probability of Type I, Type II, and Type III is lower than 6%, 7%, and 2%, respectively, in the four time periods, indicating that the possibility of local spatio-temporal correlation between the coupling coordination degree of the urbanisation level and per capita carbon emissions in the Yangtze River Economic Belt is low. In Type I, the migration probabilities of HH*_t_* → LH*_t_*_+1_, HL*_t_* → LL*_t_*_+1_, and LL*_t_* → HL*_t_*_+1_ were mostly lower than 5% (except HH*_t_* → LH*_t_*_+1_ 5.94% during 2010–2014), and the migration probability of LH*_t_* → HH*_t_*_+1_ was relatively higher, exceeding 9%. In Type II, the migration probabilities of HH*_t_* → HL*_t_*_+1_, LH*_t_* → LL*_t_*_+1_, and LL*_t_* → LH*_t_*_+1_ were mostly lower than 5% (except HL*_t_* → LH*_t_*_+1_ 7.56% during 2014–2019), and the migration probability of HL*_t_* → HH*_t_*_+1_ was mostly higher than 15% (except HL*_t_* → HH*_t_*_+1_ 7.58% during 2010–2014). In Type III, the migration probabilities of HH*_t_* → LL*_t_*_+1_ and LL*_t_* → HH*_t_*_+1_ were less than 1.5%, the migration probabilities of LH*_t_* → HL*_t_*_+1_ and HL*_t_* → LH*_t_*_+1_ were less than 5%, and the migration probability of LH*_t_* → HL*_t_*_+1_ and LL*_t_* → HH*_t_*_+1_ was 0, which reflect the extremely low probability of jump migration of the coupling coordination degree of urbanisation level and per capita carbon emissions in the Yangtze River Economic Belt.

The spatio-temporal flow (SF) and spatio-temporal condensation (SC) were calculated using the number of different types of transitions. The SC coefficients of the four time periods were all above 0.89, and the SF coefficients were all lower than 0.10, which further verified that the coupling coordination degree of the urbanisation level and per capita carbon emissions in the Yangtze River Economic Belt had strong transfer inertia. Over time, the spatio-temporal cohesion coefficient showed a fluctuating upward trend, while the SF coefficient showed a fluctuating downward trend, which reflected that the path dependence and locking characteristics of the spatial pattern of the coupling coordination degree of the urbanisation level and per capita carbon emissions in the Yangtze River Economic Belt showed a fluctuating trend. From the perspective of the migration probability of transition types, the coupling coordination degree of the urbanisation level and per capita carbon emissions in the Yangtze River Economic Belt is in a certain proportion affected by the spillover effect of neighbouring geographical units, which indicates that in addition to its own factors, the coupling coordination degree of the city is also affected by the spillover effect of the coupling coordination degree of the surrounding areas. This is mainly owing to the influence of the ‘pollution shelter effect’ and ‘pollution halo effect’. Urban per capita carbon emissions have a significant spillover effect. Urbanisation also has a strong spillover effect [60], so the coupling coordination degree of urbanisation and per capita carbon emissions shows a robust spatial spillover effect.

## 5. Discussion and Conclusions

### 5.1. Discussion

Given the differences in the key points and difficulties of urbanisation, per capita carbon emissions coupling, and collaborative development in different regions, this study highlights corresponding countermeasures and suggestions based on cities with varying urbanisation levels: (1) Accelerating the transformation of the pattern of economic growth. Cities with a high urbanisation level should take the initiative to eliminate backward production capacity, transfer labour-intensive industries to the central and western regions, and invest those resources in green and ecologically positive industries. Cities with a low urbanisation level in the central and western regions should fully use the advantages of sufficient labour endowment and actively participate in labour-intensive industries transferred from coastal areas. (2) Implementing differentiated regional carbon emissions policies. Cities with a high urbanisation level should take advantage of their favourable geographical and technological conditions and drive industrial carbon emissions reduction through technological innovation. Cities with low urbanisation levels in the central and western regions should break their dependence on the high-carbon development path rapidly. They must focus on optimising the energy structure to gradually eliminate backward industries with high energy consumption, heavy pollution, and low production capacity. (3) Strengthening cross-regional coordination mechanisms. On the one hand, we should strengthen cooperation on emissions reduction among cities in the Yangtze River Economic Belt. On the other hand, industrial planning among different regions should be further coordinated, the construction of ecological chains and ecological networks of trans-regional eco-industrial parks should be accelerated, green infrastructure cooperation should be promoted, and green and low-carbon consumption concepts should be cultivated.

Compared with previous studies, this study better describes the coupling relationship between urbanisation and per capita carbon emissions in the Yangtze River Economic Belt. The results of this study are consistent with the spatio-temporal evolution characteristics and spillover effects of regional carbon emissions in China by Zhou et al. [57] and the coupling coordination results of new urbanisation and carbon emissions in China by Jiang et al. [61]. All studies have found that the urbanisation level of the regional Yangtze River Economic Belt is continuously improving, and the carbon emission pattern has strong spatial correlation and spatial stability, mainly because China continues to promote regional coordinated development and new urbanisation. In addition, industrial development and urban development have strong path dependence, so the urbanisation pattern and carbon emission pattern are relatively stable. However, this paper uses ESTDA to analyse the degree of spatial dependence of coupling coordination and to optimise the repeated use of Moran’s *I* analysis method. The content of this study is similar to that of Song et al. [62]. Both studies found that the urbanisation of all regions is at a medium level on the whole. The difference is that the study area of this paper is the Yangtze River basin with a high level of economic development in China, whereas the study area of Song et al. [62] is mainland China, which not only includes the Yangtze River basin but also the Yangtze River Economic Belt. It also covers many regions with relatively backward economic development. The similar results may be caused by differences in urbanisation index systems. By reviewing the literature on urbanisation research, this paper selects typical indicators to represent the dimensions of population, land, and economy. The indicators are more targeted and representative and can more directly reflect the level of urban reality.

This study considers 108 cities in the Yangtze River Economic Belt as the research area and the urbanisation level and per capita carbon emissions as the research object; combines the ordination-scale law and coupled coordination degree model; discusses the coupling development law of urbanisation and per capita carbon emissions; reveals the spatio-temporal evolution characteristics of the coupled coordination degree; enriches the research means of the interaction between urbanisation level and per capita carbon emissions; deepens the research content of the interactive relationship between urbanisation level and per capita carbon emissions; and provides a reference for carbon emissions reduction work in the Yangtze River Economic Belt and other countries or regions. Although the research in this study was refined to the level of time and space, the coupling coordination relationship between urbanisation and per capita carbon emissions involved many factors. This study included only representative and accessible data indicators in the evaluation, so the selection of data indicators was insufficient. In future studies, more data should be collected to conduct a more objective evaluation of the coupling and coordination relationship between urbanisation and per capita carbon emissions.

### 5.2. Conclusions

With 108 cities in the Yangtze River Economic Belt as the research unit and urbanisation level and per capita carbon emissions in the Yangtze River Economic Belt from 2006 to 2019 as the research object, the distribution characteristics and evolution law of the coupling coordination degree of the two are analysed. The main conclusions are as follows: (1) The urbanisation level and the spatial pattern of per capita carbon emissions in the Yangtze River Economic Belt have strong stability respectively, showing a spatial pattern of ‘high in the east and low in the west’. (2) The degree of coupling coordination between urbanisation level and per capita carbon emissions in the Yangtze River Economic Belt generally declines first and then rises in time, showing a spatial distribution trend of ‘high in the east and low in the west’. The spatial pattern of the degree of coupling coordination was stable, and the spatial differences showed a decreasing trend. (3) The spatial structure of the coupling coordination degree of the urbanisation level and per capita carbon emissions in the Yangtze River Economic Belt has strong stability, dependence, and integration. The stability gradually increased from west to east, and the dependency reflected a strong spatial locking effect. The coupling coordination degree has strong transfer inertia, and the path dependence and locking characteristics of the spatial pattern of the coupling coordination degree show a trend of decreasing fluctuation over time.

## Figures and Tables

**Table 1:** Type of space–time transition.

Type	Spatio-Temporal Leap Form	Symbolic Representation
Type I	Self-leap—neighbourhood stability	HH*_t_* → LH*_t_*_+1_, LH*_t_* → HH*_t_*_+1_, LL*_t_* → HL*_t_*_+1_, HL*_t_* → LL*_t_*_+1_
Type II	Self-stability—neighbourhood leap	HH*_t_* → HL*_t_*_+1_, LH*_t_* → LL*_t_*_+1_, LL*_t_* → LH*_t_*_+1_, HL*_t_* → HH*_t_*_+1_
Type III	Self-leap—neighbourhood leap	HH*_t_* → LL*_t_*_+1_, LH*_t_* → HL*_t_*_+1_, LL*_t_* → HH*_t_*_+1_, HL*_t_* → LH*_t_*_+1_
Type IV	Self-stability—neighbourhood stability	HH*_t_* → HH*_t_*_+1_, LH*_t_* → LH*_t_*_+1_, LL*_t_* → LL*_t_*_+1_, HL*_t_* → HL*_t_*_+1_

**Table 2:** Local Moran’s *I* transition probability matrix of the coupling coordination between urbanisation level and per capita carbon emissions in the Yangtze River Economic Belt.

Time Period	*t*/*t*+1	HH	LH	LL	HL	Type	Quantity	Proportion	SF	SC
From 2006 to 2010	HH	0.9015	0.0394	0.0099	0.0493	Type Ⅰ	23	0.0532	0.1042	0.8935
	LH	0.1294	0.8353	0.0353	0.0000	Type Ⅱ	22	0.0509		
	LL	0.0100	0.0100	0.9600	0.0200	Type Ⅲ	4	0.0093		
	HL	0.1818	0.0227	0.0455	0.7500	Type Ⅳ	383	0.8866		
From 2010 to 2014	HH	0.9307	0.0594	0.0000	0.0099	Type Ⅰ	21	0.0486	0.0880	0.9120
	LH	0.0976	0.8902	0.0122	0.0000	Type Ⅱ	17	0.0394		
	LL	0.0000	0.0377	0.9528	0.0094	Type Ⅲ	0	0.0000		
	HL	0.2381	0.0000	0.0000	0.7619	Type Ⅳ	394	0.9120		
From 2014 to 2019	HH	0.8718	0.0427	0.0128	0.0726	Type Ⅰ	27	0.0500	0.1185	0.8704
	LH	0.0992	0.8264	0.0496	0.0248	Type Ⅱ	37	0.0685		
	LL	0.0084	0.0756	0.8992	0.0168	Type Ⅲ	10	0.0185		
	HL	0.0758	0.0455	0.0455	0.8333	Type Ⅳ	466	0.8630		
From 2006 to 2019	HH	0.8998	0.0469	0.0078	0.0454	Type Ⅰ	71	0.0506	0.1047	0.8903
	LH	0.1076	0.8472	0.0347	0.0104	Type Ⅱ	76	0.0541		
	LL	0.0062	0.0431	0.9354	0.0154	Type Ⅲ	14	0.0100		
	HL	0.1513	0.0263	0.0329	0.7895	Type Ⅳ	1243	0.8853		

## Data Availability

The datasets used and analysed in the current study are available from the corresponding author upon reasonable request.
